
JOURNAL INFORMATION
==============================
NLM Title Abbreviation: J Child Orthop
Journal ID: J Child Orthop
NLM Journal ID: 101313582

Journal of Children's Orthopaedics

ISSN: 1863-2521
EISSN: 1863-2548
Publisher: SAGE Publications

ARTICLE INFORMATION
==============================
PMCID: PMC5145844
DOI: 10.1007/s11832-016-0794-9
Article ID: 794
Article version: 1
Subjects: Acknowledgement

Acknowledgement to reviewers 2016

Electronic publication date: 2016 Nov 8
Print publication date: 2016 Dec
Volume: 10
Issue: 6
First page: 715
Last page: 716
Copyright: © The Author(s) 2016
License: This article is distributed under the terms of the Creative Commons Attribution 4.0 International License (http://creativecommons.org/licenses/by/4.0/), which permits unrestricted use, distribution, and reproduction in any medium, provided you give appropriate credit to the original author(s) and the source, provide a link to the Creative Commons license, and indicate if changes were made.
License URL: https://creativecommons.org/licenses/by/4.0/

issue-copyright-statement: © The Author(s) 2016

==============================

The Editors-In-Chief would like to thank the following reviewers for their continuous support during the year of 2016:

Franck Accadbled
Elizabeth Ackerman
Michael D. Aiona
Zaid Al-Aubaidi
Javier Albinana
Cristina Alves
Antonio Andreacchio
Kornelia Babin
Elhanan Bar-On
Jeremy Bauer
James Beaty
Michael J. Bell
Mohan V. Belthur
Liat Ben-Sira
Michael K. D. Benson
Ernesto Bersusky
Victor Bialik
Rainer Georg Biedermann
John G. Birch
Robert Dale Blasier
Stephanie Boehm
Silvio Boero
Daniel Brandenberger
Reinald Brunner
Carlo Camathias
Robert Campbell
Federico Canavese
Norris C. Carroll
Nelson Cassis
Pablo Castañeda
Anthony Catterall
Henry Chambers
Jack C. Y. Cheng
Tae-Joon Cho
Leonel Copeliovitch
Aurélien Courvoisier
Juan Carlos Couto
Alvin H. Crawford
Robert J. Cummings
Peter J. Cundy
Jaroslaw Czubak
Charles d'Amato
Aina J. Danielsson
Barry Danino
Luciano Dias
Matthew B. Dobbs
Deborah Eastwood
Eric W. Edmonds
John Emans
G. Ulrich Exner
Eli Ezra
Guy Fabry
David S. Feldman
Michael Fishkin
Edilson Forlin
Franz Frei
Steven Frick
Patricia M. B. Fucs
Toshio Fujii
Julia F. Funk
Rudolf Ganger
Yael Gelfer
Ismat Ghanem
Werner Girsch
Uri Givon
Christophe Glorion
Yair Gortzak
Reinhard Graf
H. Kerr Graham
Franz Grill
Robert Grimer
Richard H. Gross
Gunnar Hägglund
Chiaki Hamanishi
Theodore Harcke
Carol Hasler
Martin Haug
Dorian Hauke
Hanne B. Hedin
Bernhard Heimkes
Yoram Hemo
William Hennrikus
John A. Herring
John E. Herzenberg
Gamal Hosny
James B. Hunter
Ivan Hvid
Ernesto Ippolito
Roger Jawish
Charles E. Johnston
Stanley Jones
Benjamin Joseph
Jean-Luc Jouve
Andre Kaelin
Sam Khamis
Lori Karol
Bernd-Dietrich Katthagen
Hidehiko Kawabata
Derek M. Kelly
Paula M. Kelly
David Keret
Joseph Ivan Krajbich
Andreas H. Krieg
Ken N. Kuo
Erica Lamprecht
Mario Lampropulos
Pierre Lascombes
David Lebel
Wallace Lehman
Ruth Lester
David G. Little
Kevin Little
Randall Loder
Scott J. Luhmann
William G. Mackenzie
Hans M. Manner
Johannes Mayr

Terence McGuire
Charles T. Mehlman
A. Ludwig Meiss
Freeman Miller
Peter Miny
Bjarne Moeller-Madsen
Guy Molenaers
Jose Morcuende
Vincent Mosca
Scott J. Mubarak
Kishore Mulpuri
Marcus Mumme
Robert F. Murphy
Yasuharu Nakashima
Marek Napiontek
Cornelia Neuhaus
Peter O. Newton
Yrjänä Nietosvaara
Iulian Nusem
Hakan Omeroglu
Norman Y. Otsuka
Dror Ovadia
Klaus Parsch
Tomas Pesl
Peter Pizzutillo
Renata Pospischill
Jan Poul
Thomas Pressel
Tamir Pritsch
George T. Rab
Ehud Rath
Andreas Roposch
Erich Rutz
Ralph Sakkers
Peter B. Salamon
Wudbhav N. Sankar
Juergen Schaefer
Dietrich Schlenzka
Perry L. Schoenecker
Eitan Nathan Segev
Dalia A. Sepúlveda
David L. Skaggs
Theddy Slongo
Hae Ryong Song
Bernhard M. Speth
Carl L. Stanitski
Walter M. Strobl
Daniel Studer
Ralf Stuecker
Michael D. Sussman
Marek Synder
Terje Terjesen
Tim Theologis
Stephen J. Tredwell
Christian Tschauner
Cosimo Tudisco
Anja Van Campenhout
Johannes A. van der Sluijs
Elke Viehweger
José B. Volpon
Thekla von Kalle
Andrew Wainwright
Stewart Walsh
Daniel Weber
John H. Wedge
Dennis S. Weiner
Stuart L. Weinstein
Dennis R. Wenger
Bettina Westhoff
Roger F. Widmann
Kaye E. Wilkins
R. Baxter Willis
Thomas Wirth
James G. Wright
Haruhisa Yanagida
Moshe Yaniv
Muharrem Yazici
Reinhard D. Zeller
Kai Ziebarth

Open Access

This article is distributed under the terms of the Creative Commons Attribution 4.0 International License (http://creativecommons.org/licenses/by/4.0/), which permits unrestricted use, distribution, and reproduction in any medium, provided you give appropriate credit to the original author(s) and the source, provide a link to the Creative Commons license, and indicate if changes were made.

